# Does Obesity Predispose Medical Intensive Care Unit Patients to Venous Thromboembolism despite Prophylaxis? A Retrospective Chart Review

**DOI:** 10.1155/2016/3021567

**Published:** 2016-11-23

**Authors:** Bradley J. Peters, Ross A. Dierkhising, Kristin C. Mara

**Affiliations:** Mayo Clinic, Rochester, MN, USA

## Abstract

*Background*. Obesity is a significant issue in the critically ill population. There is little evidence directing the dosing of venous thromboembolism (VTE) prophylaxis within this population. We aimed to determine whether obesity predisposes medical intensive care unit patients to venous thromboembolism despite standard chemoprophylaxis with 5000 international units of subcutaneous heparin three times daily.* Results*. We found a 60% increased risk of venous thromboembolism in the body mass index (BMI) ≥ 30 kg/m^2^ group compared to the BMI < 30 kg/m^2^ group; however, this difference did not reach statistical significance. After further utilizing our risk model, neither obesity nor mechanical ventilation reached statistical significance; however, vasopressor administration was associated with a threefold risk.* Conclusions*. We can conclude that obesity did increase the rate of VTE, but not to a statistically significant level in this single center medical intensive care unit population.

## 1. Introduction

Obesity is a significant issue in the United States with nearly 2/3rds of the population considered to be overweight or obese and 1 in 20 adults are considered extremely obese (BMI 40 kg/m^2^+) [1]. Since the 1960s, the prevalence has doubled, but in the past 10+ years this has stabilized [2]. Globally, obesity has doubled since 1980 with 12% of the global population considered obese [3]. In our own institution, intensive care unit (ICU) patients presenting with a BMI >35 kg/m^2^ have increased every year during a 10-year period and these patients now account for over 20% of our medical ICU population. Obesity can alter many of the aspects involved in the care of these patients, from diagnostics and monitoring to medication pharmacokinetics and dosing [4]. As critical care providers, we are often in a position of extrapolating data from a standardized population and applying it to our patients. Often these standardized populations exclude or have minimal subjects in the extremes of body weight. Evidence is increasing in the care of obese patients and it is becoming more evident that obesity is going to be a common comorbidity in the general population. Much of the evidence with regard to medication variables and dosing is related to antimicrobial dosing. There is less evidence directing our interventions to prevent VTE in the medical intensive care unit (MICU) patient. In fact, the last two editions of the Antithrombotic Therapy and Prevention of Thrombosis Guidelines mention obesity as a risk factor for VTE, however leaving no recommendations regarding altered or standard chemoprophylactic dosing regimens to prevent VTE [5–7].

What is known is that critical illness alone can increase the incidence of VTE compared to a non-MICU hospitalized patient [8]. VTE is an important complication of critical illness in that it is often unrecognized while leading to sudden episodes of hypotension, bradycardia, and hypoxia, in addition to problems weaning from respiratory assistance in the case of pulmonary embolism (PE) [8]. Without VTE chemoprophylaxis, the incidence of VTE ranges from 20 to 40% in the critically ill patient population [9–13]. In 2005, Cook et al. found a 10% incidence of VTE in a medical and surgical population after 3 days with surveillance ultrasounds and a BMI of 27.1 (± 7) despite twice daily subcutaneous heparin [8]. Further evidence within mixed medical and surgical intensive care unit populations suggests potentially inadequate VTE protection with standard chemoprophylaxis dosing of subcutaneous heparin and low molecular weight heparins (LMWH) [14–17] when looking at antifactor Xa activity, although there is no established standard efficacy level.

Extrapolating data from the bariatric surgery population would seem to be a reasonable next step. Upon review of these data, there is some difficulty extrapolating from this population for the purposes of critical care as these patients are often under significantly less inflammatory stress (e.g., sepsis) and are mobilized earlier than a standard medical critical care patient. Additionally, the evidence from these trials ranges from conclusions suggesting no chemoprophylaxis is needed with early mobilization and mechanical devices to altered subcutaneous heparin and LMWH regimens being superior to standard dosing [18–26]. Given the relative lack of specific evidence based VTE chemoprophylactic dosing recommendation in a MICU population, we undertook an evaluation of our subcutaneous heparin prophylactic dosing.

## 2. Materials and Methods

We performed a single center retrospective chart review of a MICU patient population with the purpose of determining whether there is a difference in the prevalence of VTE found in patients with a BMI <30 kg/m^2^ and a BMI ≥30 kg/m^2^ while receiving (or up to 8 hours after) standard VTE chemoprophylaxis. The study was reviewed and approved by the Institutional Review Board. Inclusion criteria included the first hospitalization of all MICU patients admitted to a medical ICU aged 18 years or older who stayed in the ICU for at least 48 hours and were started on subcutaneous heparin 5000 international units three times daily (SQH) or dalteparin 5000 international units subcutaneously daily for at least 48 consecutive hours. Of note, there was a formulary change for our institution's LMWH of choice from dalteparin to enoxaparin during this time frame data; however, due to minimal use of either dalteparin or enoxaparin, this is unlikely to have affected the results. Patients were excluded if they met any of the following criteria: being pregnant and having active therapeutic treatment for confirmed or suspected VTE (warfarin, LMWH, heparin, fondaparinux, and direct thrombin inhibitors), with disease states requiring anticoagulation; history of heparin induced thrombocytopenia (HIT); or arterial thrombosis. Patients were included in the setting of atrial fibrillation so long as they were not receiving therapeutic anticoagulation. Patients were included in situations when acute coronary syndrome, pulmonary embolism, or deep vein thrombosis was in the admission differential and therapeutic anticoagulation was active until these conditions were ruled out. However, their time on therapeutic anticoagulation was not included in the data.

Our primary outcome was the rate of VTE in patients with a BMI <30 kg/m^2^ and patients with a BMI ≥30 kg/m^2^ while on standard VTE chemoprophylaxis. We tested for a null hypothesis of no difference in the rate of VTE between patients with a BMI <30 kg/m^2^ and patients with a BMI ≥30 kg/m^2^. Secondarily, we assessed VTE risk factors and characterized the patients who had a VTE.

For baseline characteristics, means and standard deviations were calculated for continuous data, and *t*-tests were used to compare obesity groups. Frequencies and percentages were computed for nominal data and Pearson's chi-square or Fisher's exact test were used as appropriate to compare obesity groups. For the primary outcome of VTE, follow-up started at subcutaneous heparin initiation and ended at 8 hours after heparin ended. A Cox proportional hazards model was constructed to compare obese to nonobese patients. A multivariable Cox proportional hazards model was also used to control for variables potentially associated with the occurrence of a VTE. Thirty patients were observed with a VTE, which allowed us to detect a minimum hazard ratio (HR) of 2.8 between the obesity groups with 80% power using a two-sided, alpha = 0.05 test, assuming equal group sizes.

## 3. Results

**Figure 1:**
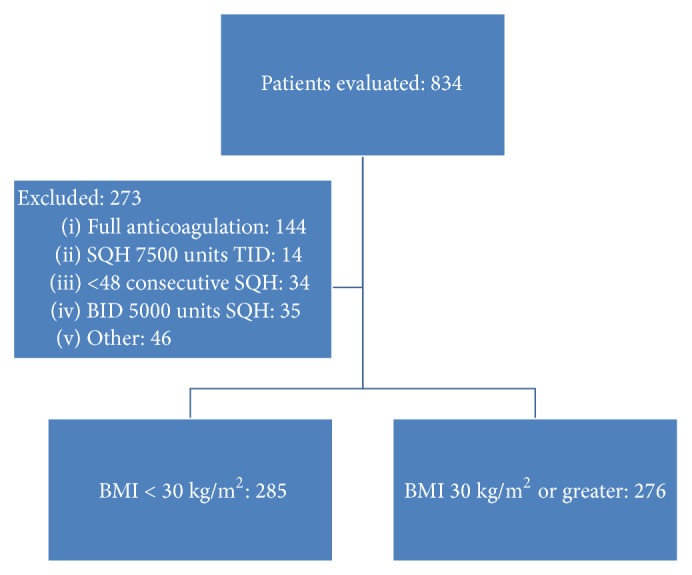
Inclusion and exclusion.

**Figure 2:**
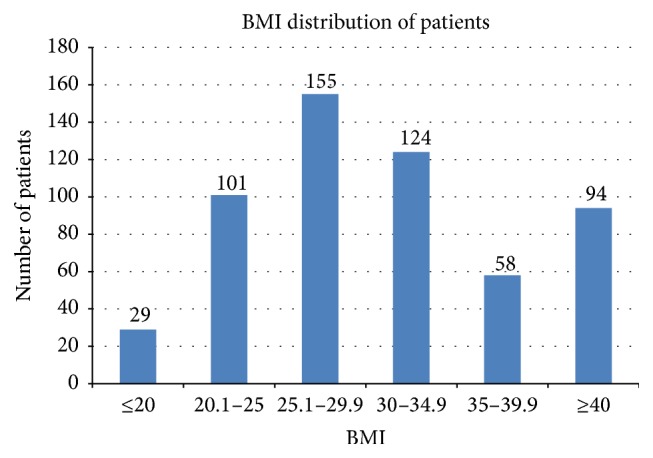
BMI distribution.

In total, 834 hospitalizations were reviewed with admission dates ranging from July 1, 2010, to July 27, 2013. After exclusion criteria were applied, 273 hospitalizations were excluded, leaving 561 patients for evaluation. Exclusion criteria are shown in Figure 1. At baseline, the groups were similar except that there was a higher percentage of males and those on a mechanical VTE prophylaxis device in the BMI <30 kg/m^2^ group. The higher mechanical VTE prophylaxis device rate is likely associated with issues of placing these devices on patients at the extremes of body habitus. More patients were admitted with a diagnosis of sepsis in the ≥30 kg/m^2^ group. Additionally, the ≥30 kg/m^2^ group had a higher percentage of patients utilizing antiplatelet agents and with documented obstructive sleep apnea (OSA). Baseline characteristics are shown in Table 1. There was no difference in the percentage of patients who received noninvasive, invasive, or a combination of the modes of ventilation. The BMI distribution was primarily in the 25–35 kg/m^2^ range. BMI distribution is shown in Figure 2. In the <30 kg/m^2^ BMI group, nearly 90% of the patients had a BMI 20–30 kg/m^2^ and in the ≥30 kg/m^2^ BMI group one-third of the patients had a BMI >40 kg/m^2^.

**Figure 3:**
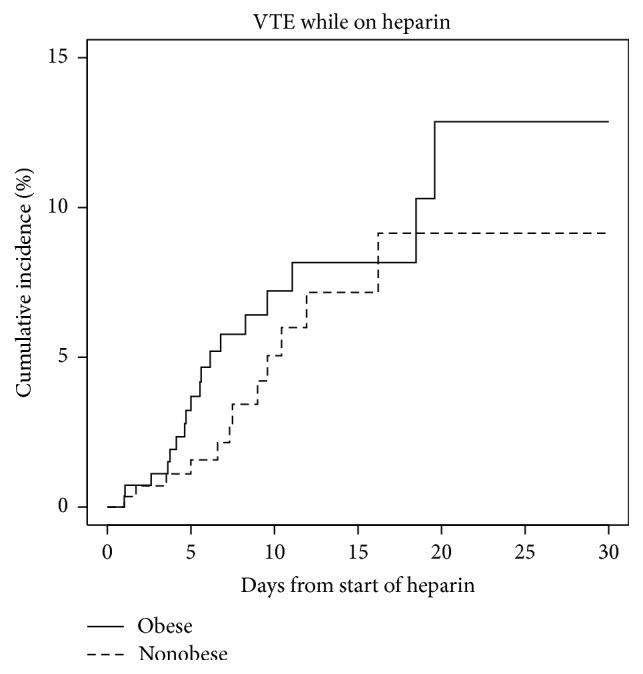
KM curve.

The primary outcome of venous thromboembolism occurred during follow-up in 12 patients in the BMI <30 kg/m^2^ group versus 18 patients in the BMI ≥30 kg/m^2^ group. The unadjusted VTE HR comparing the obese to nonobese patients was 1.596 (*p* = 0.22, CI (confidence interval) 0.75–3.38). Outcomes are listed in Table 2. With 30 events, we fit a multivariable model with 3 variables: obesity, mechanical ventilation, and vasopressors. Risk factors for VTE are listed in Table 3. Obesity and mechanical ventilation were not statistically significant (resp., HR = 1.49, *p* = 0.2971, CI 0.7022–3.180; HR = 1.06, *p* = 0.9164, CI 0.3647–3.074). Vasopressor use had a HR 3.037 (*p* = 0.0316, CI 1.1028–8.364) suggesting a tripling of risk. A VTE was more likely to happen within the first 7 days for the higher BMI group. Kaplan-Meier (KM) curve is shown in Figure 3. The BMI <30 kg/m^2^ group had all observed events occurring within 17 days of taking heparin, with an incidence of 2.2% in the first 7 days. In comparison, VTE events for the BMI 30 kg/m^2^ or greater group all occurred within 20 days with an incidence of 5.8% in the first 7 days. Most of the observed VTE events happened in the ICU setting (24/30) and a majority of the VTE events were DVTs (21/30). There was no difference in the rate of bleeding during hospitalization with 27 events in the BMI <30 kg/m^2^ group versus 26 events in the BMI ≥30 kg/m^2^ group (HR = 1.074, *p* = 0.8031, CI 0.612–1.887). Sixty deaths occurred during the hospitalization in the BMI <30 kg/m^2^ group, versus 37 in the BMI ≥30 kg/m^2^ group.

## 4. Discussion

Despite finding an approximately 60% increase risk of VTE in medical ICU patients with a BMI ≥30 kg/m^2^ compared to a BMI <30 kg/m^2^ with VTE chemoprophylaxis, this did not reach statistical significance. Due to the low incidence of VTE events, only 3 covariates could be used in our risk model. From this model, obesity and mechanical ventilation were still not statistically significant; however, the use of vasopressors was associated with a threefold increased risk. This appears to be consistent with findings in prior VTE chemoprophylactic literature in other populations, suggesting vasopressors alter the absorption and distribution of a subcutaneously administered product [8, 27].

Looking at the characteristics of patients who developed VTE during follow-up, we noticed that events were grouped around the middle range of BMI (25–35 kg/m^2^). Characteristics of VTE are listed in Table 4. Proportionally, there were fewer males with an observed VTE event in the BMI ≥30 kg/m^2^ group but they were similar in age. The majority of patients had a respiratory process driving their reason for admission and this could be expected as our MICU is primarily run by critical care pulmonologists and there are separate neurologic and cardiovascular ICUs. The acute physiology score (APS) is similar between the groups suggesting minimal differences in severity of illness. Most of the patients were on a vasopressor at some point in the ICU stay, but 2 patients in the BMI <30 kg/m^2^ group were on vasopressors at the time of the event. In the BMI ≥30 kg/m^2^ group, 3 patients were on active vasopressors at the time of the event. Proportionally, more patients in the BMI ≥30 kg/m^2^ group had a prior diagnosis of OSA or active hematologic or oncologic malignancy. None of the patients who had an observed VTE event were chronic dialysis patients. More patients in the BMI ≥30 kg/m^2^ group were on an antiplatelet agent, which should have some additional protective effect against thrombosis. Due to the low event rate, this was not included in our risk model; however, in assessing the characteristics of patients who developed a VTE, antiplatelet agents were present in a quarter of the BMI <30 kg/m^2^ group and a third of the BMI ≥30 kg/m^2^ group suggesting that antiplatelet agents would likely not alter our results significantly. Heparin induced thrombocytopenia was found in 2 patients in each group.

A recent evaluation explored the preventability of VTE [28]. According to these data, there are VTE events that are unlikely to be preventable. These are primarily associated with catheter-associated deep vein thrombosis (DVT). When looking at our data, during follow-up, the BMI <30 kg/m^2^ group had 6 catheter-associated DVTs and the BMI ≥30 kg/m^2^ group had 3 catheter-associated DVTs. Based on Haut's definition of an unpreventable DVT, half of the VTE events in the lower BMI group were not preventable, whereas only a sixth of the events in the higher BMI group would be considered not preventable. This information points at a more significant effect of obesity if nonpreventable VTE could be removed from the results.

In a recent analysis of thrombosis predictors in a randomized thromboprophylaxis trial enrolling patients with elevated BMI, with a family or personal history of VTE, those receiving vasopressors were at the greatest risk [27]. The increased risk of VTE with vasopressors was similar to our findings. In contrast, we did not show the statistically significant risk associated with BMI although this could be related to our lower-than-predicted incidence compared to our use of the PROTECT study to guide our incidence. The PROTECT study was much larger and screened for DVT prospectively with surveillance ultrasound. Additionally, the PROTECT study utilized dalteparin 5000 units daily or subcutaneous heparin 5000 units twice daily (BID). Our evaluation utilized a more aggressive SQH dosing regimen and involved few patients receiving low molecular weight heparin for chemoprophylaxis. Despite these differences, both of these studies add to the growing evidence that increased BMI and the use of vasopressors may increase the risk of VTE in critically ill patients.

The main limitations of this study are that it was retrospective and our power calculations assumed a higher incidence of VTE events than what was observed. In previous prospective studies, surveillance ultrasounds were utilized to look for VTE (primarily DVT) on a regular basis and could find possible asymptomatic VTE, thus increasing the event rate. Since we conducted a chart review, we were dependent on clinical suspicion and symptomatic VTE and this likely reduced our incidence rate. As demonstrated from prior autopsy results that critically ill patients may have VTE with minimal or no symptoms, a protocol utilizing surveillance ultrasounds would likely produce more events [29]. Many prior studies evaluating the incidence of VTE in the critically ill patients were also multicenter studies greatly increasing the power for detecting a lower frequency event with chemoprophylaxis. Given the low rate of VTE while on chemoprophylactic measures, we overestimated our event rate. Our findings showed a non-statistically significant increase of VTE risk in the BMI ≥30 kg/m^2^ sample while receiving standard chemical prophylactic measures. Furthermore, we aimed to capture LMWH in this evaluation but due to minimal patients receiving one of these agents our results cannot be applied to this class of chemotherapeutic agents. A larger multicenter study that can recruit more patients for evaluation is likely needed to find a difference and this would preferably be performed in a prospective fashion. Future studies should evaluate whether the relationship between BMI and VTE risk is linear and if there is a transition point at which the VTE risk significantly rises. This would allow clinicians to better identify patients at a higher risk of VTE events. Additionally, they should evaluate altered dosing regimens to achieve clinical or surrogate end points associated with a reduction of VTE events. A possible surrogate end point could be an antifactor Xa activity range associated with reduced risk.

## 5. Conclusions

Obesity is increasing not only in the United States but across the global population. We as critical care clinicians should prepare for the potential differences this population may present in their care. VTE chemoprophylaxis is a standard of care for critically ill patients; however, prior literature points to possible shortfalls in the protection of the obese population from VTE events while undergoing ICU level of care. The thousands of dollars of additional cost to diagnose, bed, and treat a new VTE add to the importance of adequately protecting critically ill patients from this consequence [30]. In this single center retrospective review, we found a higher incidence of VTE in the BMI ≥30 kg/m^2^ group compared to the BMI <30 kg/m^2^ group, although this did not reach statistical difference. This study highlights the need for further study of VTE prevention in the obese population, in particular whether standard dosing or altered dosing of prophylactic agents is required.

## Figures and Tables

**Table 1:** Baseline demographics.

	BMI < 30 kg/m^2^	BMI ≥ 30 kg/m^2^	*p*
	*n* = 285	*n* = 276	
Male (%)	159 (56%)	126 (46%)	0.016
Average BMI (SD), kg/m^2^	24.8 (3.3)	38.8 (8.8)	
Age (SD)	61.8 (16.9)	64.2 (14.2)	0.074
Primary reason for admission			0.014
Cardiovascular	22 (7.7%)	14 (5.1%)	
Respiratory	151 (53%)	133 (48%)	
Gastrointestinal	10 (3.5%)	19 (6.9%)	
Renal	7 (2.5%)	11 (4%)	
Neurologic	19 (6.7%)	8 (2.9%)	
Sepsis	50 (17.5%)	74 (26.8%)	
Metabolic	6 (2.1%)	5 (2%)	
Other	20 (7%)	12 (4.3%)	
Active hematologic or oncologic process	50 (17.5%)	35 (12.7%)	0.108
Documented sleep apnea	20 (7%)	85 (30.8%)	<0.001
History of thrombosis	15 (5.3%)	20 (7.2%)	0.332
Treatment of thrombosis	15 (100%)	19 (95%)	
complete			
Antiplatelet agents			0.0021
Aspirin	89 (31.2%)	119 (43%)	
Clopidogrel	1 (0.5%)	4 (1.5%)	
Both	9 (3.2%)	14 (5.1%)	
1-hour acute physiology score (SD)	36 (19.5)	38.5 (20.1)	0.14
24-hour acute physiology score (SD)	62.8 (21.4)	66 (21.5)	0.075
Dialysis			0.203
Chronic	15 (5.3%)	18 (6.5%)	
New/acute	26 (9.1%)	37 (13.4%)	
Mechanical ventilation			0.119
Noninvasive	45 (15.8%)	65 (23.6%)	
Invasive	112 (39.3%)	92 (33.3%)	
Both	72 (25.3%)	65 (23.6%)	
IVC filter	11 (3.9%)	7 (2.5%)	0.374
Mechanical VTE prophylaxis device	119 (41.7%)	88 (31.3%)	0.015
Subcutaneous heparin	282 (98.9%)	270 (97.8%)	
Subcutaneous dalteparin	3 (1.1%)	6 (2.2%)	

**Table 2:** Outcomes.

Outcomes	BMI < 30 kg/m^2^	BMI ≥ 30 kg/m^2^	Unadjusted hazard ratio (95% CI), obese versus nonobese	*p*
VTE	12	18	1.596 (0.7537–3.381)	0.222
VTE in ICU	10	14		
DVT	9	12		
PE	0	2		
Both	1	0		
VTE during rest of hospitalization	2	4		
DVT	2	3		
PE	0	1		
Both	0	0		

**Table 3:** Risk factors for VTE.

Risk factors for VTE	Hazard ratio (95% CI)	*p*
Obesity	1.494 (0.7022–3.180)	0.2971
Vasopressor use	3.037 (1.1028–8.364)	0.0316
Mechanical ventilation	1.059 (0.3647–3.074)	0.9164

**Table 4:** Characteristics of VTE patients.

Characteristics	BMI < 30 kg/m^2^	BMI ≥ 30 kg/m^2^
	*N* = 12	*N* = 18
BMI of patients with VTE (SD)	25.7 (3.23)	39.3 (9.7)
Less than or equal to 20	1	0
20.1–25	3	0
25.1–29.9	8	0
30–34.9	0	9
35–39.9	0	3
Greater than or equal to 40	0	6
Male (%)	6 (50%)	7 (39%)
Age (SD)	61.5 (15.4)	63 (15.1)
Primary admission Dx		
Cardiovascular	0	3
Respiratory	8	8
Gastrointestinal	0	3
Renal	1	1
Neurologic	1	0
Sepsis	2	3
Other	0	0
Secondary admission Dx		
Cardiovascular	0	1
Respiratory	2	2
Gastrointestinal	0	2
Renal	1	1
Neurologic	0	0
Sepsis	3	2
Other	1	0
APS, 1 hour (SD)	42 (31.5)	39.2 (19.3)
APS, 24 hours (SD)	70.5 (17.6)	68.4 (24.2)
On vasopressor anytime	10	12
Vasopressor at time of event	2	3
Norepinephrine	1	3
Vasopressin	1^a^	0
Epinephrine	0	0
Phenylephrine	1^a^	0
Dopamine	0	0
Dobutamine	0	0
Antiplatelet agent	3	6
Prior thrombosis	2	2
Sleep apnea	1	5
Active oncologic/hematologic malignancy	3	9
Noninvasive ventilation at any time prior to event	5	9
Noninvasive ventilation at time of event	0	2
Invasive ventilation at any time prior to event	10	13
Invasive ventilation at time of event	8	8
Dialysis		
Acute/new	3	6
Chronic	0	0
IVC filter	1	3
Mechanical VTE prophylaxis documented	7	7
HIT	2	2

^a^Same patient on all vasopressors.
